# Attosecond dynamics of multi-channel single photon ionization

**DOI:** 10.1038/s41467-022-32780-5

**Published:** 2022-09-03

**Authors:** Jasper Peschel, David Busto, Marius Plach, Mattias Bertolino, Maria Hoflund, Sylvain Maclot, Jimmy Vinbladh, Hampus Wikmark, Felipe Zapata, Eva Lindroth, Mathieu Gisselbrecht, Jan Marcus Dahlström, Anne L’Huillier, Per Eng-Johnsson

**Affiliations:** 1 Department of Physics, Lund University, P.O. Box 118, 22100 Lund, Sweden; 2 Physikalisches Institut, Albert-Ludwigs-Universität, Stefan-Meier-Strasse 19, 79104 Freiburg, Germany; 3 Department of Physics, Stockholm University, AlbaNova University Center, SE-106 91 Stockholm, Sweden

**Keywords:** Attosecond science, Quantum mechanics

## Abstract

Photoionization of atoms and molecules is one of the fastest processes in nature. The understanding of the ultrafast temporal dynamics of this process often requires the characterization of the different angular momentum channels over a broad energy range. Using a two-photon interferometry technique based on extreme ultraviolet and infrared ultrashort pulses, we measure the phase and amplitude of the individual angular momentum channels as a function of kinetic energy in the outer-shell photoionization of neon. This allows us to unravel the influence of channel interference as well as the effect of the short-range, Coulomb and centrifugal potentials, on the dynamics of the photoionization process.

## Abstract (web summary)

Understanding of photoionization dynamics, one of the fastest processes in nature, requires the characterization of all underlying ionization channels. Here the authors use an interferometry technique based on attosecond pulses to measure the phase and amplitude of the individual angular momentum channels in the photoionization of neon.

## INTRODUCTION

Photoionization is a fundamental process that happens when electromagnetic radiation of high enough frequency is absorbed by matter. In general, an electron is released into the continuum via various ionization channels corresponding to different initial or final angular momenta. The reconstruction of photoionization dynamics requires determination of the complex-valued amplitudes of the different ionization channels.

Since the 70s, synchrotron radiation has been used for pioneering studies^1^, playing an important role in our understanding of the quantum nature of matter and its interaction with light. The importance of angle-resolved measurements^2,3^ to investigate the respective contribution of angular-momentum channels has been understood for a long time, and experiments have been performed on a number of atomic and molecular systems^4,5^. Advances towards the characterization of the complex-valued ionization channel amplitudes^6–8^ have been achieved by determining the electron spin^9,10^, or alternatively measuring (or preparing) the residual ion alignment and orientation with sophisticated experimental protocols including coincidence techniques^11^ and/or the combination of light fields with several polarization components^12–14^. As a result of these decades of efforts, two recent studies using molecular frame photoelectron angular distribution imaging have succeeded to measure single-photon^15,16^ angle-resolved phases in molecules up to an isotropic but energy-dependent phase.

The temporal reconstruction of photoionization dynamics requires, however, to determine the phase variation with energy for all involved channels and not only the phase difference between channels which add coherently. Experimentally, this requires coherent measurements over a large energy range in order to access the ultrafast, attosecond, dynamics, and angular detection, to analyze the interference between different outgoing angular momentum channels.

Two-photon schemes combining either laser-generated attosecond extreme ultraviolet (XUV) pulses^17–20^ or free-electron laser femtosecond pulses^21^ with infrared (IR) laser pulses have opened up a new path towards the full temporal reconstruction of photoionization dynamics. Through the variation of this two-photon interaction with the delay between the two pulses, energy-resolved phase difference measurements can be performed, often by interferometric techniques^22–24^. Angle-resolved detection gives information on the emission angle dependence^17,25–28^. The fact that a second photon is essential to these methods means that a great amount of complexity is added to the measurement. The interaction with the IR field adds an extra phase and projects the electron wavepacket on different angular momentum states, so that the characterization of one-photon ionization becomes difficult. In practice, these measurements have been limited to one (dominant) ionization channel, enabling partial access to temporal information such as the Wigner time delay, i.e. the derivative of the scattering phase, for a given angular momentum^29–31^, or to the characterization of the final two-photon wavepacket^32^.

In this article, we characterize the one-photon ionization dynamics from the 2*p*^6^-ground state of neon, obtaining the amplitude and phase of the electric dipole transition matrix elements towards *ϵs*- and *ϵd*-continuum states. To extract channel-resolved one-photon ionization phases, we extend the angle-resolved RABBIT (reconstruction of attosecond beating by interference of two-photon transitions) technique^18,25,27,28,32^ by using a multi-channel analysis of the photoelectron angular distribution (PAD), while making use of the universality of the continuum-continuum transitions. We then determine the channel-resolved one-photon amplitudes by using the extracted phases for the analysis of single-photon ionization PADs. The retrieved values for the one-photon phases and amplitudes are in excellent agreement with calculations using angular-channel-resolved many-body perturbation theory^33–35^. Since we determine the energy variation of channel-resolved phases (in contrast to phase differences between channels), we are able to fully reconstruct the spatial and temporal dynamics of the photoionization process, including the coherent or incoherent contributions from the different channels, as well as the effect of the short-range, Coulomb and centrifugal potentials.

## Results and discussion

### Single photon ionization

**Fig. 1:**
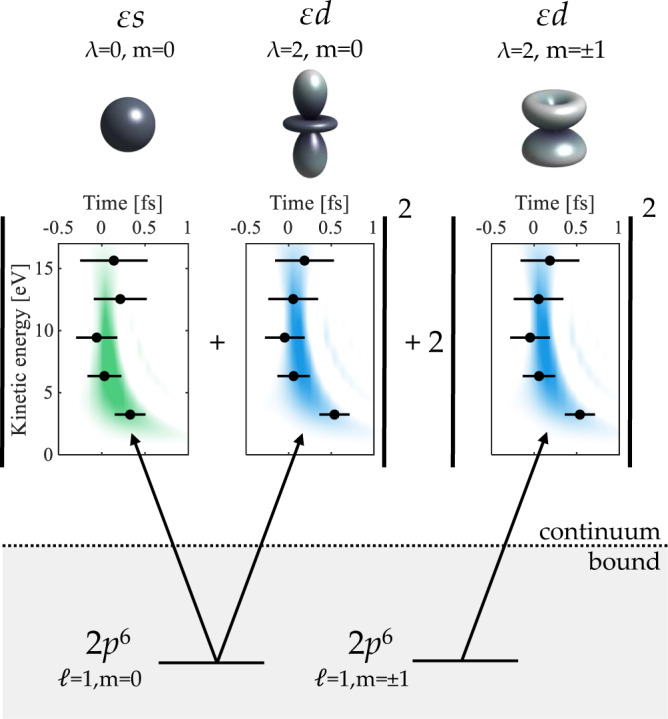
Single-photon ionization of the 2*p*^6^-ground state of neon. Illustration of the photoionization process for the angular momentum channels, 2p → *ε*s, m = 0, 2p → *ε*s d, m = 0 2p → *ε*d m = ± 1. A time-energy representation (the Wigner distribution) of the created broadband wavepackets for each channel, based on simulations, is shown in green (2p → *ε*s) and blue (2p → *ε*d). The data points represent our experimental measurements of Wigner time delays. The channels with different initial states add incoherently, while those with the same initial state and different final states add coherently, as indicated by the mathematical signs. The angular distributions for each channel, given by spherical harmonics, are indicated at the top.

Figure 1 illustrates photoionization from the 2*p*^6^-ground state of neon using linearly-polarized light. The ionization signal can be written as an incoherent sum over different initial states characterized by the magnetic quantum numbers *m* = 0, ± 1. For *m* = 0, two different angular momentum channels, 2*p* → *εs* and 2*p* → *εd*, add coherently. In general, the theoretical description of photoionization is model dependent, and within the dipole approximation, far from resonant regions and Cooper minima, relativistic effects can be neglected. The complex amplitudes then reduce to the single-particle amplitudes^10^. Each channel is characterized by a matrix element *M*_*λℓm*_, *ℓ* = 1 denoting the initial orbital quantum number and *λ* = 0, 2 the final angular momentum. The one-photon angle-dependent photoelectron signal can be expressed as:

1 $${I}_{{{\mbox{H}}}}(\theta )\propto {\left|{M}_{010}{Y}_{00}(\theta,\phi )+{M}_{210}{Y}_{20}(\theta,\phi )\right|}^{2}+2{\left|{M}_{211}{Y}_{21}(\theta,\phi )\right|}^{2},$$

where *Y*_*λm*_ are spherical harmonics, depending on the polar and azimuthal angles *θ* and *ϕ*. Note that *I*_H_ does not depend on *ϕ*, since ${Y}_{\lambda m}(\theta,\phi )\propto \exp (im\phi )$. The absolute values of the spherical harmonics involved in the single photoionization of Ne are shown at the top of Fig. 1. The matrix elements are complex-valued quantities given by

2 $${M}_{\lambda \ell m} \,\approx\, {C}_{\lambda \ell }^{m}\,{a}_{\lambda \ell }\,\exp \left(\,{{\mbox{i}}}\,{\varphi }_{\lambda \ell }\right),$$

where *a*_*λℓ*_ represent the one-photon amplitudes, *φ*_*λℓ*_ the one-photon phases and ${C}_{\lambda \ell }^{m}$ known angular coefficients [See Eq. (1) in Supplementary Note 1]. The phase *φ*_*λℓ*_ is accumulated by the electron on its trajectory in the atomic potential and thus includes a contribution from the short range, centrifugal and Coulomb potentials. Knowing the matrix elements as a function of energy, it is possible to retrieve the temporal dynamics, as indicated in Fig. 1 and demonstrated in this article.

### Experimental results

**Fig. 2:**
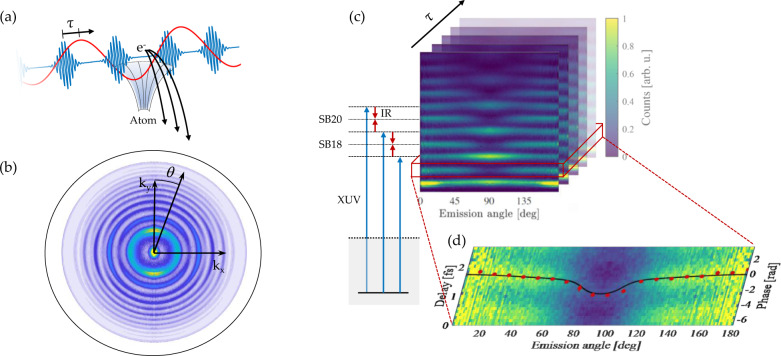
Illustration of our experimental procedure. **a** XUV pulses (blue) and IR field (red) with a delay *τ* release photoelectrons via a two-photon transition. **b** Delay-integrated momentum distribution (*k*_*x*_, *k*_*y*_) of the released two-photon electron wavepacket at an emission angle *θ*. **c** Illustration of the experimental technique, where angle-resolved two-photon photoelectron spectrum are recorded as a function of delay *τ* (black arrow). Also indicated are the different pathways leading sidebands 18 and 20 after the interaction with XUV (blue) and IR photons (red). For the sake of statistics, we show the delay-integrated photoelectron spectra. **d** Energy-integrated sideband 16 as a function of angle and delay. The red dots show the extracted oscillation phase, and the black line shows the result of a simulation (see Methods section).

To this end, we apply the two-photon RABBIT technique with full angular resolution, as illustrated in Fig. 2a, b. XUV pulses from high-order harmonic generation (HHG)^36^ in argon in the 20-50 eV range and weak IR pulses from the laser driving the HHG process interact with a gas of neon atoms. Our experiment consists in recording the photoelectron momentum distributions [Fig. 2b] with a velocity-map imaging spectrometer (see^37,38^) as a function of delay between the XUV and IR pulses (see experimental details in the Methods section). The extracted photoelectron spectra consist of main peaks due to absorption of the 15^th^ to 25^th^ harmonics and sidebands involving an additional absorption or emission of an IR photon. These sidebands can be reached by two interfering quantum paths [Fig. 2c]^18,39^ and thus oscillate as a function of delay at a frequency equal to 2*ω*, where *ω* is the angular frequency of the driving laser. Figure 2d shows the delay and angle dependence of sideband 16 integrated over an energy range of 0.8 eV around the peak. The oscillation phase is extracted by fitting a cosine to the temporal evolution for each angle. The red dots in Fig. 2d show the measured oscillation phase as a function of the emission angle. The phase variation as a function of angle is the strongest close to 80^∘^ and 100^∘^ where it jumps by ∼2 rad. In contrast to previous measurements of similar phase shifts^27,28,40,41^, the high XUV pulse energy in our experiment allows us to observe sideband signal at all emission angles, even at 90^∘^ where the signal amplitude is low. The experimental data is compared to simulations (black line) based on calculations presented in the Methods section.

### Phase retrieval

The angle- and delay-dependent sideband intensity can be written as

3 $${I}_{SB}(\theta,\tau )\,\,\propto \,\,\mathop{\sum}\limits_{m=0,\pm 1}\,\,{\bigg|\mathop{\sum}\limits_{\begin{array}{c}\scriptstyle L=1,3\\ \scriptstyle \lambda=0,2\end{array}}{Y}_{Lm}(\theta,\phi )\left({{{{{{{{\mathcal{M}}}}}}}}}_{L\lambda \ell m}^{+}{{{\mbox{e}}}}^{{{\mbox{i}}}\omega \tau }+{{{{{{{{\mathcal{M}}}}}}}}}_{L\lambda \ell m}^{-}{{{\mbox{e}}}}^{-{{\mbox{i}}}\omega \tau }\right) \bigg|}^{2}\,\,,$$

where ( ± ) refers to the pathways with IR absorption ( + ) or emission ( − ), ${{{{{{{{\mathcal{M}}}}}}}}}_{L\lambda \ell m}^{\pm }$ is the two-photon transition matrix element with final, intermediate and initial angular momentum *L*, *λ* and *ℓ* respectively. The initial magnetic quantum number *m* is kept constant in the two-photon transition, since the XUV and IR fields are linearly polarized in the same direction. The phase term e^±i*ωτ*^, which arises from the interaction with the IR field, depends on the delay *τ* between the two pulses. The two-photon transition matrix element for sideband 2*q* can be decomposed into amplitude and phase terms as

4 $${{{{{{{{\mathcal{M}}}}}}}}}_{L\lambda \ell m}^{\pm }={C}_{L\lambda }^{m}{C}_{\lambda \ell }^{m}\,{a}_{L\lambda \ell }^{\pm }\,\exp \left[\,{{\mbox{i}}}\,\left({\phi }_{L\lambda }^{\pm }+{\varphi }_{\lambda \ell,2q\mp 1}+{{{\Phi }}}_{2q\mp 1}\right)\right].$$

Here, ${a}_{L\lambda \ell }^{\pm }$ is the two-photon radial amplitude. The phase term includes three different contributions: ${\phi }_{L\lambda }^{\pm }$ arises from the transition between continuum states^31,41,42^ and is therefore called "continuum-continuum” (cc) phase, *φ*_*λℓ*,2*q*∓1_ is the phase associated to the one-photon ionization channel *ℓ* → *λ* after absorption of harmonic order 2*q* ∓ 1, and Φ_2*q*∓1_ is the phase of the (2*q*∓1)^th^ harmonic field. For the cc-phase and the two-photon radial amplitude ${a}_{L\lambda \ell }^{\pm }$, the superscript denotes absorption ( + ) or emission ( − ) of an IR photon.

As seen in Eq. (3) and Eq. (4), the angular structure of the sidebands does not only depend on the interference between different angular momentum channels reached via single photon ionization. It is also strongly influenced by the measurement involving an additional transition with the IR field, which leads to different (two-photon) transition amplitudes, a change of parity of the spherical harmonics and additional phases ${\phi }_{L\lambda }^{\pm }$.

Our method for extraction of the single-photon phases *φ*_*λℓ*,2*q*∓1_ is based on the fact that, far enough from resonances, cc transitions are to a large extent insensitive to the short range potential and only depend on the Coulomb potential, making them universal quantities (see Supplementary Note 2). The relative amplitude and phase of these transitions have been studied in great detail theoretically^31,35,40,43^ and experimentally^42^. Using the calculations described in the Methods section, it is possible to reduce the number of unknown parameters in Eq. (3) (see Supplementary Note 3) to a point where they can be determined from the experiment through the following fitting procedure.

We perform an angular analysis of the experimental data based on an expansion of *I*_SB_ into Legendre polynomials^5,41,42,44^, as

5 $${I}_{{{\mbox{SB}}}}(\theta,\tau )={h}_{0}(\tau ){P}_{0}(\,{{\mbox{cos}}}\,\,\theta )+{h}_{2}(\tau ){P}_{2}(\,{{\mbox{cos}}}\,\,\theta )+{h}_{4}(\tau ){P}_{4}(\,{{\mbox{cos}}}\,\,\theta ),$$

where *P*_*i*_ (*i* = 0,2,4) are polynomials of order 0, 2 and 4 (*P*_0_ = 1) and *h*_*i*_(*τ*) the coefficients of the expansion. For a two-photon transition, without parity mixing, the expansion needs only three polynomials, and as shown in Supplementary Note 3, their coefficients *h*_*i*_(*τ*) oscillate with delay at frequency 2*ω*. Combining Eqs. (3) and (5), we can express the coefficients in terms of the unknown parameters (see eqs. (4)–(6) in the Supplementary Note 3), and the phases can then be determined using a fitting procedure, which is repeated for each sideband. The single-photon phases extracted from two consecutive sidebands are identical for the intermediate harmonic order. We can thus reference all the extracted phases to a single, global, absolute phase offset. It is worth noting that we thereby determine not only the phase difference between channels as a function of energy, as can be done in a single photon experiment at e.g. a synchrotron, but also the phase variation with energy for both channels, which is necessary for the reconstruction of the temporal dynamics.

### Amplitude retrieval

Since the absorption or emission of infrared photons redistributes the photoelectrons from the main band to the sideband, thus modifying the angular distribution, the one-photon amplitudes need to be extracted from measurements in the absence of the IR field. *I*_H_(*θ*), defined in Eq. 1, can be written as an expansion of Legendre polynomials *P*_0_ and *P*_2_ [see Supplementary Note 3]. Similarly to the two-photon case, using the one-photon phases obtained previously, we determine the radial amplitudes of the *λ* = 0 and *λ* = 2 channels up to a common, global factor.

### Channel-resolved amplitudes and phases

**Fig. 3:**
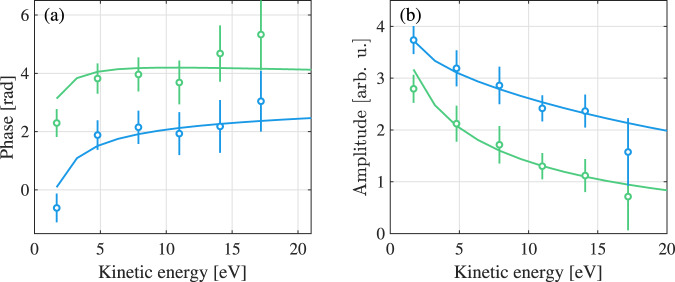
One-photon phases and amplitudes as a function of the kinetic energy. **a** Experimental (dots) and calculated (solid line) one-photon phases *φ*_01_ (green) and *φ*_21_ (blue). The error bars correspond to one standard deviation. **b** Radial amplitudes *a*_21_ and *a*_01_ extracted from the single-photon data (dots) and simulated data (solid lines). The error bars indicate the standard deviation as explained in Supplementary Note 4.

Figure 3 presents the one photon phases *φ*_*λℓ*_ (a) and amplitudes *a*_*λℓ*_ (b) as a function of energy for *λ* = 0 (green) and 2 (blue). In the remainder of the article, we disregard the index 2*q* ∓ 1 of the single-photon phases. The experimentally retrieved data are indicated as circles, while results of calculations, described in the method section, are shown as solid lines. In (a), we adjust the global phase offset and in (b) a global amplitude factor to give the best agreement between theory and experiment. The observed deviation of the lowest energy might be due to an interference with a resonant pathway due to absorption of harmonic 13 and two IR photons^45–48^. The phases of both angular channels increase similarly with energy, differing by ∼*π*. The amplitudes decrease with energy. The ratio between *a*_21_ and *a*_01_ is above one at all energies, in agreement with Fano’s propensity rule for one-photon absorption^49^.

**Fig. 4:**
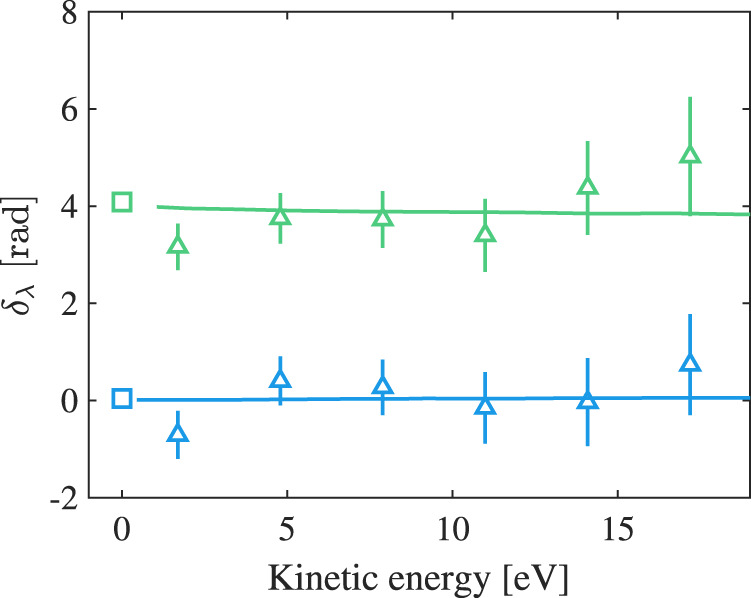
Phase shifts induced by the short-range potential. Retrieved phase contribution from the short-range potential *δ*_*λ*_ for the s- and d-channel (green and blue respectively) as a function of kinetic energy. The contributions from the Coulomb phase and the centrifugal potential are subtracted from the experimentally retrieved one-photon phases (triangles) and compared to calculations from Kennedy and Manson^50^ (solid lines) as well as predictions from quantum defect theory at threshold (squares). The error bars indicate the standard deviation as explained in Supplementary Note 4.

The results shown in Fig. 3a allow us to study the different contributions to the phase, in particular the short range (correlation) effects. The phase *φ*_*λℓ*_ can be written as the sum of the scattering phase *η*_*λ*_ and a contribution from the centrifugal barrier − *πλ*/2^31^. The scattering phase is itself the sum of the Coulomb phase ${\varsigma }_{\lambda }=\arg {{\Gamma }}(\lambda+1-iZ/k)$ and a contribution *δ*_*λ*_ from the short range potential. Here *Z* is the atomic number, $k=\sqrt{2{m}_{e}E}/\hslash$ the wavenumber, *m*_*e*_ denoting the electron mass and *E* its kinetic energy, and Γ, the gamma function. In Fig. 4 we show *δ*_0_ and *δ*_2_ (green and blue triangles) obtained from the experimental data by subtracting the Coulomb phase and the effect of the centrifugal barrier (− *π* for *λ* = 2). The short range effect induces a phase shift which does not vary much over the studied energy range, and which is significant (∼1.2*π*) only for the s-electron since the centrifugal barrier prevents the d-electron to be close to the core. These results agree well with calculations from Kennedy and Manson^50^ (solid lines). Finally, we calculate the quantum defect for s and d Rydberg states using tabulated data^51^ and take the limit for high energies. According to scattering theory^52,53^, this asymptotic quantum defect *μ*_*λ*_ multiplied by *π* is equal to the short range phase at threshold. The obtained phases, indicated as squares in Fig. 4, agree with the experimental and theoretical data.

### Spatial and temporal dynamics

The energy variation of channel-resolved phases allows us to describe the spatial and temporal dynamics of the single photon ionization process. Photoionization of neon in the 2p^6^ shell by absorption of a (single) broadband XUV attosecond pulse creates coherent wavepackets *ψ*_*λm*_(*t*)*Y*_*λm*_(*θ*, *ϕ*) in each angular momentum channel,

6 $${\psi }_{\lambda m}(t){Y}_{\lambda m}(\theta,\phi )\, =	 \int dE\,{{{{{{{{\mathcal{E}}}}}}}}}_{{{\mbox{XUV}}}}(E){C}_{\lambda \ell }^{m}\,{a}_{\lambda \ell }(E){Y}_{\lambda m}(\theta,\phi ) \\ 	 \exp \left\{-\,{{\mbox{i}}}\,\left[\frac{Et}{\hslash }-{\varphi }_{\lambda \ell }(E)\right]\right\},$$

where ${{{{{{{{\mathcal{E}}}}}}}}}_{{{\mbox{XUV}}}}$ represents the envelope of the XUV pulse. We show a time-frequency representation of these channel-resolved contributions in Fig. 1, using a Wigner distribution, defined as

7 $${W}_{\lambda m}(E,t)=\int d\tau \,{\psi }_{\lambda m}^{*}\left(t+\frac{\tau }{2}\right){\psi }_{\lambda m}\left(t-\frac{\tau }{2}\right)\exp \left(\frac{\,{{\mbox{i}}}\,E\tau }{\hslash }\right).$$

Note that for *λ* = 2, the representations are equal for *m* = 0 and *m* = ± 1, apart for a scaling factor given by the angular coefficients ${C}_{\lambda \ell }^{m}$. The distributions represented in color in Fig. 1 (green for *λ* = 0 and blue for *λ* = 2) are obtained from the calculations assuming a transform-limited, broadband, attosecond XUV pulse. The experimentally retrieved Wigner time delays, defined as Δ*φ*_*λℓ*_/2*ω*, where Δ*φ*_*λℓ*_ is the difference between two consecutive phases, are indicated as dots. This representation illustrates the ultrafast dynamics of the photoionization process, which take place during a few hundreds of attoseconds. The negative chirp of the electron wavepacket, with the low energy electrons being delayed relative to the high energy ones, is mostly due to the energy dependence of the Coulomb phase for both angular momentum channels. Dynamical effects due to correlation are negligible in this case.

**Fig. 5:**
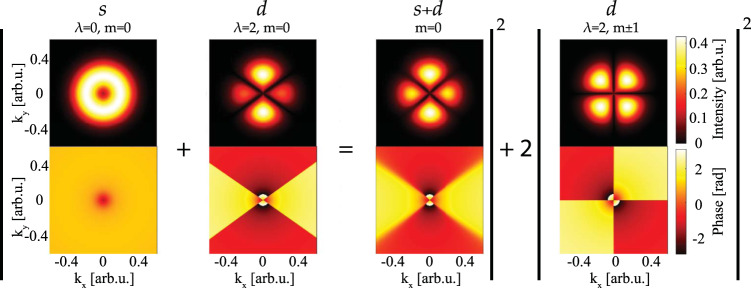
Channel-resolved electron wavepacket. Amplitude (top row) and phase (bottom row) of the electron wavepackets as a function of *k*_*x*_ and *k*_*y*_ for a given time. Shown are the individual channels as well as the coherent sum of the *λ* = 0 and *λ* = 2 channels for the case of *m* = 0 (third column).

In order to illustrate the spatial properties of the electron wavepacket, we show in Fig. 5 a snapshot of the momentum distribution. The amplitude and the phase of the integrand of Eq. (6) are shown as a function of the wave vector components *k*_*x*_ and *k*_*y*_, for the different channels. For *λ* = 2 the wavepacket exhibits angular nodes at the magic angles (54.7^∘^, 125.3^∘^) for *m* = 0 and (0^∘^, 90^∘^) for *m* = ± 1 where the amplitude is zero and the phase jumps by *π*. We also show in the third column of Fig. 5 the coherent sum of the s- and d-channels. Their interference leads to modification of the momentum distribution with an enhancement of the features at 90^∘^ and a continuous phase variation across the angular nodes. It is worth noting that this reconstruction requires the knowledge of the energy variation of the channel-resolved one-photon phases and not only the phase difference.

### Conclusion

In summary, our multi-channel analysis of two-photon PADs allows us to determine the one-photon phases and amplitudes as a function of energy for each angular momentum channel. Both the energy dependence and the difference between the angular channels is well reproduced by calculations. The retrieval is demonstrated from the 2*p*^6^ ground state of neon, but our method is general and can be applied to other initial shells (e.g. *d*) and thus to more complex atomic systems. The study of the phases of the different angular momentum channels unravels the interplay between short-range, correlation and/or centrifugal effects. In addition, our measurements enable the reconstruction of the full dynamics of the ionized wavepacket in time, space and momentum.

## Methods

### Experimental setup

The XUV attosecond pulse trains (APTs) used in the experiments are synthesized using high-order harmonic generation (HHG) by focusing 40 fs, 806 nm, ∼45 mJ pulses from a 10 Hz Ti:sapphire laser into a pulsed gas jet of argon in a loose focusing geometry ( ∼8.7 m focal length)^54^. The obtained XUV spectrum spans from ∼20 to 50 eV. The beamline is designed to generate high-flux high-order harmonics up to 1 *μ*J, hence the loose focusing geometry^55^.

**Fig. 6:**
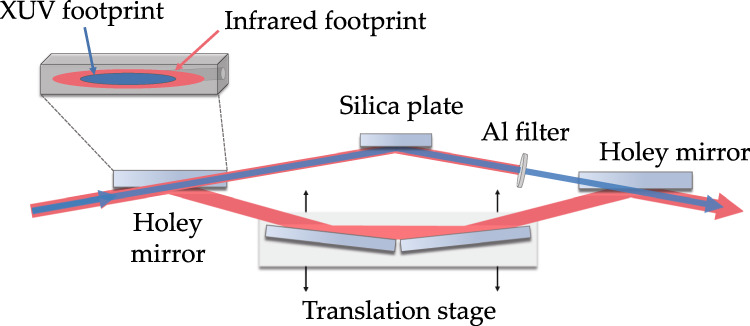
Schematic drawing of the XUV-infrared interferometer. Two holey mirrors split and recombine the XUV and infrared components of the beam and a linear translation stage introduces a time delay between them.

The APTs are separated from the fundamental infrared field by taking advantage of the lower divergence of the XUV beam. Figure 6 shows a scheme of the newly developed interferometer, which consists of a set of holey mirrors creating two interferometric arms. The hole diameter is chosen in a way such that the entire XUV beam propagates through the hole, whereas the reflected part only consists of fundamental infrared radiation. A translation stage in the infrared arm varies the path difference between both arms and hence introduces a time delay. A 200 nm thick aluminum filter in the XUV arm blocks the remaining infrared and the intense low-order harmonics. Due to the long beamline design, traditional interferometers add the risk of pointing instabilities and temporal jitters, while propagating over such long distances. Hence, we designed this compact in-line interferometer, such that the path difference is kept as short as possible, which results in excellent temporal and spatial stability.

Both beams, after being collinearly overlapped, are focused tightly using two toroidal mirrors in a Wolter configuration^56^. In focus they interact with the neon target gas introduced using a pulsed Even-Lavie valve^57^ and a set of two skimmers. The resulting photoelectrons are detected by a velocity map imaging spectrometer (VMIS) with the ability to record angle-resolved momentum distribution^37,38^. The 2D projections of the momentum distribution of the photoelectrons is recorded by a CCD camera imaging a phosphor screen coupled to a set of multi-channel plates. Since the VMIS is recording the 2D projection of the 3D momentum distribution, an inverse Abel transform has to be applied, which is done using an iterative method^58^. The angular response of the detector was verified by comparing the *β*-parameters extracted from single-photon ionization angular distributions with data from Taylor et al.^59^.

### Numerical method

Our calculations are based on a one-electron Hamiltonian, with a Dirac-Fock potential plus a correction that ensures the correct long-range potential for ionized photoelectrons^33^. The absorption of one ionizing photon is treated within the Relativistic Random Phase Approximation with Exchange (RPAE) resulting in a so-called perturbed wave function describing the ionized electron. The method accounts for important many-body effects such as inter-channel coupling and ground-state correlation. Exterior complex scaling is used in order to be able to use a finite numerical box.

The complex-valued two-photon matrix elements, expressed in Eq. (4), are then calculated as the transition from the perturbed wave function to the final continuum state in each angular momentum channel, following the procedure described in^33–35^ for the non-relativistic case. The integration is performed numerically out to a distance far outside the atomic core, but within the unscaled region, while the last part of the integral is carried out using analytical Coulomb waves along the imaginary radial axis. The amplitude and phase shift of these Coulomb waves are determined from the numerical solutions for the perturbed wave function and for the final state describing a free electron within the potential of the remaining ion. The numerical stability is monitored by comparison of different "break points” between the numerical and analytical descriptions.

## Supplementary information

Supplementary Information

## Data Availability

The data of the main results, shown in Fig. 3, can be accessed via the following link: 10.5878/h5yr-gb56. Figures 4 and 5 can be derived from this data. The raw detector data is available from the authors upon request.

## Code availability

The analysis code is available from the authors upon request.
