# Supplementary material for: Attosecond dynamics of multi-channel single photon ionization
Source: Nat Commun. 2022 Sep 3;13:5205. doi: 10.1038/s41467-022-32780-5 (PMC9440915; doi:10.1038/s41467-022-32780-5)
Supplement: Supplementary file 1 — Supplementary Information [file 41467_2022_32780_MOESM1_ESM.pdf]

Supplementary Information for:

Attosecond dynamics of multi-channel single photon ionization

Jasper Peschel, David Busto, Marius Plach, Mattias Bertolino, Maria Hoflund, Sylvain Maclot, Jimmy Vinbladh, Hampus Wikmark, Felipe Zapata, Eva Lindroth, Mathieu Gisselbrecht, Jan Marcus Dahlström, Anne L'Huillier, and Per Eng-Johnsson

Supplementary Note 1: Angular coefficients The angular coefficients in the general form $C^m_{l,l+1}$ are derived by calculating the angular transition matrix element between an initial state with angular momentum ℓ and a final state with angular momentum $\ell+1$:

$$C^m_{\ell,\ell+1} = (-1)^{\ell+m-1}(\ell+1)^{1/2}\begin{pmatrix} \ell+1 & 1 & \ell \\ -m & 0 & m \end{pmatrix}. \tag{S1}$$

Supplementary Note 2: Continuum-continuum transition Applying and extending the results of previous work [1,2,3], we use the universal behavior of the cc-transitions to determine some of the terms in Eq. (3), thus reducing the number of unknown quantities.

Supplementary Figure 1 (a,d) presents the ratios between the two-photon transition radial amplitudes, calculated as described in the methods section and defined as

$$\alpha^{\pm}_{\lambda\ell} = \frac{a^{\pm}_{(\lambda-1)\lambda\ell}}{a^{\pm}_{(\lambda+1)\lambda\ell}}, \tag{S2}$$

for increasing or decreasing angular momentum from the same intermediate state as the function of the kinetic energy of the electron. The different curves correspond to different

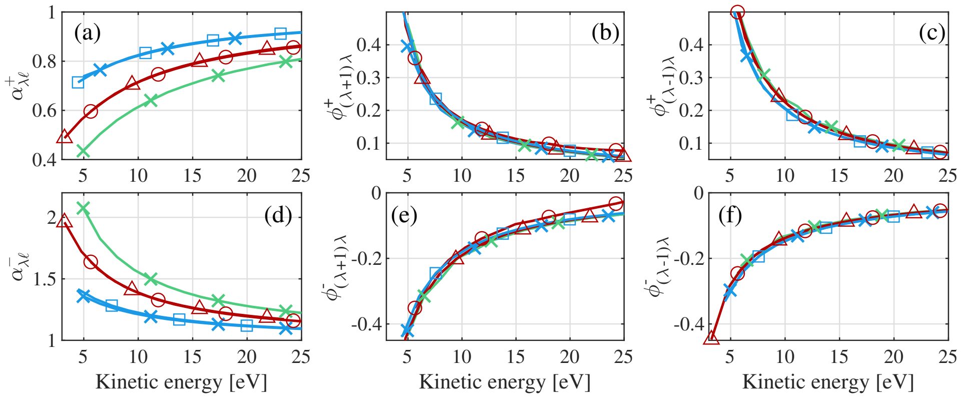

Supplementary Figure 1: Continuum-continuum transitions. Calculated amplitude ratios $\alpha^{\pm}_{\lambda\ell}$ (a,d) and continuum-continuum phases $\phi^{\pm}_{L\lambda}$ (b-c and e-f) for the absorption (a-c) and emission (d-f) processes. For the amplitude ratios, the curves correspond to different intermediate states (λ=1,2,3 in blue, red, green, respectively) and different atoms/initial states (square, He, $\ell=0$); (cross, Kr, $\ell=2$); (triangle, Ne, $\ell=1$); (circle, Ar, $\ell=1$). (b,e) refer to transitions with increasing angular momentum, $L=\lambda+1$, while in (c,f), $L=\lambda-1$.

intermediate states (λ=1,2,3 in blue, red and green) and different atoms and initial states. The ratios show an universal behavior [3], independent of the atom. Only the orbital angular momentum of the intermediate state, λ, is of importance.

We present $\phi^{\pm}_{L\lambda}$ in Supplementary Figure 1 for increasing (b,e) and decreasing (c,f) angular momenta, in the absorption (b,c) and emission cases (e,f). The colors and symbols, corresponding to different intermediate angular momenta and atoms/initial states are indicated in the figure caption. These results show that the variation of the continuum-continuum phase is universal, depending mainly on whether the IR photon is absorbed or emitted. For the absorption process, the cc-phase decreases as a function of kinetic energy and is positive, while for the emission, it increases and is negative. Note that the phases are not mirror image of each other, i.e. $\phi^{+}_{L\lambda} \neq -\phi^{-}_{L\lambda}$. The cc-phases depend on whether the angular momentum increases or decreases, especially at low kinetic energy, as observed by comparing (b) and (c), or (e) and (f). Finally, the cc-phases depend only slightly on the intermediate angular momentum (compare blue and red curves) and not at all on the atomic system (e.g. compare circle and triangle) in the range of energies studied here.

Supplementary Note 3: Phase retrieval Our channel-resolved amplitude and phase retrieval is based on the knowledge of the cc-transitions. The unknown and known quantities in Eq. (3), after having expressed the transition matrix elements as in Eq. (4), and used the available information in Supplementary Figure 1, are indicated in Table 1 for an initial $\ell = 1$ state. We note that only the phase difference $\Delta\Phi_{2q} = \Phi_{2q+1} - \Phi_{2q-1}$, and not the individual high-order harmonic phases, plays a role in Eq. (3). The number of unknown quantities is therefore nine. While here the case of $\ell = 1$ is shown, this is also true for higher initial angular momenta.

TABLE 1: Unknown and known quantities involved in Eq. (1)

	Unknown quantities	Known quantities
Atomic phases	$\varphi_{01,2q\mp1}$, $\varphi_{21,2q\mp1}$	$\phi^{\pm}_{10}$, $\phi^{\pm}_{12}$, $\phi^{\pm}_{32}$, from Supp. Fig. 1(b,c,e,f)
2-photon amplitudes	$a^{\pm}_{101}$, $a^{\pm}_{321}$	$a^{\pm}_{121} = \alpha^{\pm}_{21} a^{\pm}_{321}$<br>$\alpha^{\pm}_{21}$ from Supp. Fig. 1(a,d)
Harmonic phases	$\Delta\Phi_{2q} = \Phi_{2q-1} - \Phi_{2q+1}$	

We determine these nine unknown quantities using a global fit to our experimental measurements. In general, multiphoton electron angular distributions can be written as an expansion in Legendre polynomials [2,4,5,6]. For a two-photon transition, without parity mixing, the expansion needs only three polynomials, $P_0(x) = 1$, $P_2(x) = (3x^2 - 1)/2$, and $P_4(x) = (35x^4 - 30x^2 + 3)/8$, reading as

$$I_{\mathrm{SB}}(\theta, \tau) = h_0(\tau) + h_2(\tau) P_2(\cos\theta) + h_4(\tau) P_4(\cos\theta). \tag{S3}$$

The theoretical expressions for the coefficients $h_i(\tau)$, $i = 0, 2, 4$, can be obtained by expanding Eq. (3) and replacing the products of spherical harmonics by Legendre polynomials, leading to the following equations:

$$
\begin{aligned}
h_0(\tau) =& \frac{25}{9}\Big[(a_{101}^{+})^2 + (a_{101}^{-})^2\Big] + \frac{34}{9}\Big[(\alpha_2^{+} a_{321}^{+})^2 + (\alpha_2^{-} a_{321}^{-})^2\Big] + 4\Big[(a_{321}^{+})^2 + (a_{321}^{-})^2\Big] \\
&+ \frac{40}{9}\Big[\alpha_2^{+} a_{101}^{+} a_{321}^{+} \cos\big(\varphi_{01,2q-1} - \varphi_{21,2q-1} + \phi_{10}^{+} - \phi_{12}^{+}\big) \\
&+ \alpha_2^{-} a_{101}^{-} a_{321}^{-} \cos\big(\varphi_{01,2q+1} - \varphi_{21,2q+1} + \phi_{10}^{-} - \phi_{12}^{-}\big)\Big] \\
&+ \frac{50}{9}\; a_{101}^{+} a_{101}^{-} \cos\big(2\omega\tau + \mathit{\Delta\Phi}_{2q} + \varphi_{01,2q-1} - \varphi_{01,2q+1} + \phi_{10}^{+} - \phi_{10}^{-}\big) \\
&+ \frac{40}{9}\Big[(\alpha_2^{-} a_{101}^{+} a_{321}^{-} \cos\big(2\omega\tau + \mathit{\Delta\Phi}_{2q} + \varphi_{01,2q-1} - \varphi_{21,2q+1} + \phi_{10}^{+} - \phi_{12}^{-}\big) \\
&+ \alpha_2^{+} a_{321}^{+} a_{101}^{-} \cos\big(2\omega\tau + \mathit{\Delta\Phi}_{2q} + \varphi_{21,2q-1} - \varphi_{01,2q+1} + \phi_{12}^{+} - \phi_{10}^{-}\big)\Big] \\
&+ \frac{68}{9}\alpha_2^{+} \alpha_2^{-} a_{321}^{+} a_{321}^{-} \cos\big(2\omega\tau + \mathit{\Delta\Phi}_{2q} + \varphi_{21,2q-1} - \varphi_{21,2q+1} + \phi_{12}^{+} - \phi_{12}^{-}\big) \\
&+ 8\; a_{321}^{+} a_{321}^{-} \cos\big(2\omega\tau + \mathit{\Delta\Phi}_{2q} + \varphi_{21,2q-1} - \varphi_{21,2q+1} + \phi_{32}^{+} - \phi_{32}^{-}\big)
\end{aligned}
\tag{S4}
$$

$$
\begin{aligned}
h_2(\tau) =& \frac{50}{9}\Big[(a_{101}^{+})^2 + a_{101}^{-})^2\Big] + \frac{14}{9}\Big[(\alpha_2^{+} a_{321}^{+})^2 + (\alpha_2^{-} a_{321}^{-})^2\Big] + \frac{32}{7}\Big[(a_{321}^{+})^2 + (a_{321}^{-})^2\Big] \\
&+ \frac{80}{9}\Big[\alpha_2^{+} a_{101}^{+} a_{321}^{+} \cos\big(\varphi_{01,2q-1} - \varphi_{21,2q-1} + \phi_{10}^{+} - \phi_{12}^{+}\big) \\
&+ \alpha_2^{-} a_{101}^{-} a_{321}^{-} \cos\big(\varphi_{01,2q+1} - \varphi_{21,2q+1} + \phi_{10}^{-} - \phi_{12}^{-}\big)\Big] \\
&+ \frac{96}{7}\Big[\alpha_2^{+} (a_{321}^{+})^2 \cos\big(\phi_{32}^{+} - \phi_{12}^{+}\big) + \alpha_2^{-} (a_{321}^{-})^2 \cos\big(\phi_{32}^{-} - \phi_{12}^{-}\big)\Big] \\
&+ \frac{60}{7}\Big[a_{101}^{+} a_{321}^{+} \cos\big(\varphi_{01,2q-1} - \varphi_{21,2q-1} + \phi_{10}^{+} - \phi_{32}^{+}\big) \\
&+ a_{101}^{-} a_{321}^{-} \cos\big(\varphi_{01,2q+1} - \varphi_{21,2q+1} + \phi_{10}^{-} - \phi_{32}^{-}\big)\Big] \\
&+ \frac{100}{9} a_{101}^{+} a_{101}^{-} \cos\big(2\omega\tau + \mathit{\Delta\Phi}_{2q} + \varphi_{01,2q-1} - \varphi_{01,2q+1} + \phi_{10}^{+} - \phi_{10}^{-}\big) \\
&+ \frac{80}{9}\Big[\alpha_2^{-} a_{101}^{+} a_{321}^{-} \cos\big(2\omega\tau + \mathit{\Delta\Phi}_{2q} + \varphi_{01,2q-1} - \varphi_{21,2q+1} + \phi_{10}^{+} - \phi_{12}^{-}\big) \\
&+ \alpha_2^{+} a_{321}^{+} a_{101}^{-} \cos\big(2\omega\tau + \mathit{\Delta\Phi}_{2q} + \varphi_{21,2q-1} - \varphi_{01,2q+1} + \phi_{12}^{+} - \phi_{10}^{-}\big)\Big] \\
&+ \frac{60}{7}\Big[a_{321}^{+} a_{101}^{-} \cos\big(2\omega\tau + \mathit{\Delta\Phi}_{2q} + \varphi_{21,2q-1} - \varphi_{01,2q+1} + \phi_{32}^{+} - \phi_{10}^{-}\big) \\
&+ a_{101}^{+} a_{321}^{-} \cos\big(2\omega\tau + \mathit{\Delta\Phi}_{2q} + \varphi_{01,2q-1} - \varphi_{21,2q+1} + \phi_{10}^{+} - \phi_{32}^{-}\big)\Big] \\
&+ \frac{28}{9}\; \alpha_2^{+} \alpha_2^{-} a_{321}^{+} a_{321}^{-} \cos\big(2\omega\tau + \mathit{\Delta\Phi}_{2q} + \varphi_{21,2q-1} - \varphi_{21,2q+1} + \phi_{12}^{+} - \phi_{12}^{-}\big) \\
&+ \frac{64}{7} a_{321}^{+} a_{321}^{-} \cos\big(2\omega\tau + \mathit{\Delta\Phi}_{2q} + \varphi_{21,2q-1}\varphi_{21,2q+1} + \phi_{32}^{+} - \phi_{32}^{-}\big) \\
&+ \frac{96}{7}\Big[\alpha_2^{-} a_{321}^{+} a_{321}^{-} \cos\big(2\omega\tau + \mathit{\Delta\Phi}_{2q} + \varphi_{21,2q-1} - \varphi_{21,2q+1} + \phi_{32}^{+} - \phi_{12}^{-}\big) \\
&+ \alpha_2^{+} a_{321}^{+} a_{321}^{-} \cos\big(2\omega\tau + \mathit{\Delta\Phi}_{2q} + \varphi_{21,2q-1} - \varphi_{21,2q+1} + \phi_{12}^{+} - \phi_{32}^{-}\big)\Big]
\end{aligned}
\tag{S5}
$$

$$\begin{aligned}
h_4(\tau) =& \frac{24}{7}\Big[(a^{+}_{321})^2 + (a^{-}_{321})^2\Big] + \frac{16}{7}\Big[\alpha_2^{+}(a^{+}_{321})^2\cos\big(\phi^{+}_{32}-\phi^{+}_{12}\big) + \alpha_2^{-}(a^{-}_{321})^2\cos\big(\phi^{-}_{32}-\phi^{-}_{12}\big)\Big]\\
&+ \frac{80}{7}\Big[a^{+}_{101}a^{+}_{321}\cos\big(\varphi_{01,2q-1}-\varphi_{21,2q-1}+\phi^{+}_{10}-\phi^{+}_{32}\big)\\
&+ a^{-}_{101}a^{-}_{321}\cos\big(\varphi_{01,2q+1}-\varphi_{21,2q+1}+\phi^{-}_{10}-\phi^{-}_{32}\big)\Big]\\
&+ \frac{48}{7}\,a^{+}_{321}a^{-}_{321}\cos\big(2\omega\tau+\Delta\Phi_{2q}+\varphi_{21,2q-1}-\varphi_{21,2q+1}+\phi^{+}_{32}-\phi^{-}_{32}\big)\\
&+ \frac{16}{7}\Big[\alpha_2^{-}a^{+}_{321}a^{-}_{321}\cos\big(2\omega\tau+\Delta\Phi_{2q}+\varphi_{21,2q-1}-\varphi_{21,2q+1}+\phi^{+}_{32}-\phi^{-}_{12}\big)\\
&+ \alpha_2^{+}a^{+}_{321}a^{-}_{321}\cos\big(2\omega\tau+\Delta\Phi_{2q}+\varphi_{21,2q-1}-\varphi_{21,2q+1}+\phi^{+}_{12}-\phi^{-}_{32}\big)\Big]\\
&+ \frac{80}{7}\Big[a^{+}_{321}a^{-}_{101}\cos\big(2\omega\tau+\Delta\Phi_{2q}+\varphi_{21,2q-1}-\varphi_{01,2q+1}+\phi^{+}_{32}-\phi^{-}_{10}\big)\\
&+ a^{+}_{101}a^{-}_{321}\cos\big(2\omega\tau+\Delta\Phi_{2q}+\varphi_{01,2q-1}-\varphi_{21,2q+1}+\phi^{+}_{10}-\phi^{-}_{32}\big)\Big] \qquad \text{(S6)}
\end{aligned}$$

The coefficients $h_i(\tau)$ are extracted from the data by a fit of equation S3 to the background subtracted and normalized photoelectron angular distributions (PADs) for each delay. Supplementary Figure 2 shows the variation of $h_i(\tau)$ extracted from the experimental data for each delay (black points). As is clear from Eqs. (S4-S6) and from Supplementary Figure 2, each $h_i(\tau)$ oscillates with the delay τ at the frequency 2ω and is therefore fully determined by three quantities: mean value, amplitude and phase. Thus, a total of nine parameters describe the angle and delay dependence of the sideband signal $I_{\mathrm{SB}}(\theta,\tau)$. This implies that the nine unknown quantities in table 1 can be determined through a global fit of the three analytical expressions of $h_i(\tau)$, to the experimentally measured coefficients in Supplementary Figure 2. This simultaneous fit to the derived system of equations is based on a Levenberg-Marquardt algorithm using a least square regression and extensively tested for its convergence using theoretical data. The result of such a global fit is shown by the red lines in Supplementary Figure 2.

The nine unknown quantities in table 1 are not completely independent, since the one-photon ionization phases only appear as differences in Eqs. (S4-S6). We therefore lock $\varphi_{01,15}$ for the first harmonic order (15) and determine the other three phases involved in this measurement ($\varphi_{21,15}$, $\varphi_{01,17}$, $\varphi_{21,17}$). The global fit is repeated for the next sideband (18), which involves a contribution from the same harmonic order (17) as sideband 16. Using the previously determined phases for harmonic 17, we then retrieve the one-photon phases for harmonic 19. This process is iteratively repeated over the entire energy range allowing us to map out the energy dependence of the one-photon phases.

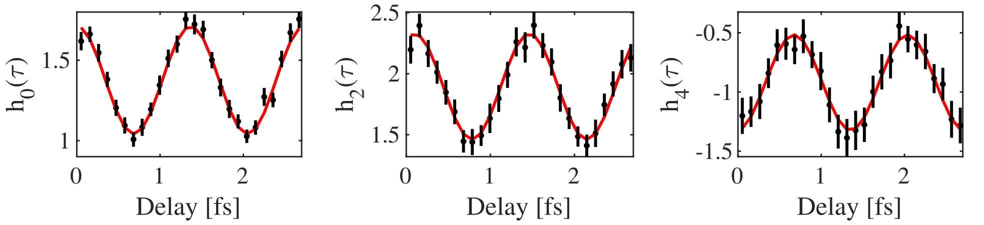

Supplementary Figure 2: Global fit to experimental data. Delay dependence of the coefficients $h_i(\tau)$, $i = 0, 2, 4$ for sideband 18. The black dots are obtained from the angular distributions (Eq. S3) for each delay and the error bars correspond to one standard deviation. The red curves are the result of a simultaneous fit of the delay dependent h_i functions using Eqs. (S2-S4) in the SM. The error bars indicate the standard deviation extracted from the fit.

The fit allows the retrieval of the one-photon phases, the two-photon amplitudes and the harmonic phase difference. However, since only the one-photon ionization is studied in this work, only the one-photon phase is considered.

Supplementary Note 4: Error estimation The error bars of the extracted phases are calculated in two steps: First, the standard deviation returned from the fit of equation (S3) to the PADs is extracted. Second, the global fit of S4-S6 is performed 500 times, using values for each $h_i(\tau)$ normally distributed around the result of the fit and with a width equal to the standard deviation determined in the first step. The standard deviation of the resulting values for φ_{01}, φ_{21} is then used for the error bars in figure 4.

Supplementary Note 5: Amplitude retrieval $I_{\mathrm{H}}(\theta)$ can be written as an expansion of Legendre polynomials P_0 and P_2, where the two expansion coefficients can be written as:

$$h_0 = \frac{1}{12\pi}\Big[a_{01}^2 + a_{21}^2\Big], \tag{S7}$$

$$h_2 = \frac{1}{3\pi}\Big[\frac{1}{2}a_{21}^2 + a_{01}a_{21}\,\cos(\varphi_{01} - \varphi_{21})\Big]. \tag{S8}$$

The coefficients of the expansion h_0 and h_2 are extracted from the experimental data. Using the one-photon phases obtained previously, we determine the relative radial amplitudes of the $\lambda = 0$ and $\lambda = 2$ channels.

Supplementary References

1. J Marcus Dahlström, Diego Guénot, Kathrin Klünder, Matthieu Gisselbrecht, Johan Mauritsson, Anne L'Huillier, Alfred Maquet, and Richard Taïeb. Theory of attosecond delays in laser-assisted photoionization. *Chem. Phys.*, 414:53–64, 2013.
2. Jaco Fuchs, Nicolas Douguet, Stefan Donsa, Fernando Martin, Joachim Burgdörfer, Luca Argenti, Laura Cattaneo, and Ursula Keller. Time delays from one-photon transitions in the continuum. *Optica*, 7(2):154–161, Feb 2020.
3. David Busto, Jimmy Vinbladh, Shiyang Zhong, Marcus Isinger, Saikat Nandi, Sylvain Maclot, Per Johnsson, Mathieu Gisselbrecht, Anne L'Huillier, Eva Lindroth, and Jan Marcus Dahlström. Fano's propensity rule in angle-resolved attosecond pump-probe photoionization. *Phys. Rev. Lett.*, 123:133201, 2019.
4. J Joseph, F Holzmeier, D Bresteau, C Spezzani, T Ruchon, J F Hergott, O Tcherbakoff, P D'Oliveira, J C Houver, and D Dowek. Angle-resolved studies of XUV–IR two-photon ionization in the RABBITT scheme. *Journal of Physics B: Atomic, Molecular and Optical Physics*, 53(18):184007, aug 2020.
5. Katharine L Reid. Photoelectron angular distributions. *Annu. Rev. Phys. Chem*, 54(1):397–424, 2003.
6. E. Arnous, S. Klarsfeld, and S. Wane. Angular distribution in the two-quantum atomic photoeffect. *Phys. Rev. A*, 7:1559–1568, May 1973.
